# Aging Microglia—Phenotypes, Functions and Implications for Age-Related Neurodegenerative Diseases

**DOI:** 10.3389/fnagi.2017.00194

**Published:** 2017-06-14

**Authors:** Björn Spittau

**Affiliations:** ^1^Department of Molecular Embryology, Faculty of Medicine, Institute for Anatomy and Cell Biology, University of FreiburgFreiburg, Germany; ^2^Institute of Anatomy, University of RostockRostock, Germany

**Keywords:** aging, microglia, neurodegeneration, Alzheimer’s disease (AD), Parkinson’s disease (PD)

## Abstract

Aging of the central nervous system (CNS) is one of the major risk factors for the development of neurodegenerative pathologies such as Parkinson’s disease (PD) and Alzheimer’s disease (AD). The molecular mechanisms underlying the onset of AD and especially PD are not well understood. However, neuroinflammatory responses mediated by microglia as the resident immune cells of the CNS have been reported for both diseases. The unique nature and developmental origin of microglia causing microglial self-renewal and telomere shortening led to the hypothesis that these CNS-specific innate immune cells become senescent. Age-dependent and senescence-driven impairments of microglia functions and responses have been suggested to play essential roles during onset and progression of neurodegenerative diseases. This review article summarizes the current knowledge of microglia phenotypes and functions in the aging CNS and further discusses the implications of these age-dependent microglia changes for the development and progression of AD and PD as the most common neurodegenerative diseases.

## Introduction

Microglia are the resident immune cells of the central nervous system (CNS) and are involved in a multifaceted range of physiological as well as pathophysiological functions (Prinz and Priller, 2014). Recent scientific efforts have resulted in the elucidation of the developmental origin, the molecular signature and the versatile functions of these macrophage-like glia cells and gained insight into their nature. Especially, the developmental origin of microglia and the associated phenomenon that these CNS-specific immune cells underlie a self-propelling regeneration causing telomere shortening has resulted in the hypothesis that microglia age, develop age-dependent cellular dystrophy and become senescent (Streit, 2006). Microglial senescence has been linked to functional changes that are high likely to contribute to an age-dependent increase of microglia-mediated neuroinflammatory responses, which are believed to further threaten aged neurons and, thus, drive the progression of age-related neurodegenerative pathologies, such as Alzheimer’s disease (AD) and Parkinson’s disease (PD). This review article summarizes the current knowledge of functional and phenotypic properties of aged microglia and highlights the contribution of aged microglia to the development and progression of AD and PD.

## Microglia Origin

The origin of microglia has long been debated and initial studies suggested that microglia differ substantially from perivascular and meningeal macrophages, which were described to derive from blood-borne monocytes and are constantly replenished (Hickey and Kimura, 1988; Bechmann et al., 2001). Recent reports have demonstrated that both, CNS-specific macrophages and microglia do not develop from circulating monocytes. CNS macrophages including perivascular macrophages, meningeal macrophages and to a lesser extent choroid plexus macrophages arise from hematopoietic precursors during embryonic development in a PU.1-dependent manner and further establish stable cell populations that do not experience turnover and replacement from bone-marrow-derived cells (Goldmann et al., 2016). The developmental origin of microglia has been revealed by elegant fate-mapping experiments, which clearly demonstrated that PU.1 and Irf8-dependent pathways are crucial for microglial embryonic development (Kierdorf et al., 2013). Moreover, this development from yolk sac progenitors is independent of IL-34. However, microglia migration towards and population of the CNS parenchyma relies on neuronal expression and secretion of IL-34, which serves as the most potent homing factor for microglia precursors (Greter et al., 2012). In order to sense IL-34, the microglial expression of colony stimulating factor-1 receptor (CSF1R) is required. Interestingly, the presence of CSF1 itself is not necessary for proper microglia development and colonization of the CNS (Ginhoux et al., 2010). The development of sophisticated methods to immediately isolate microglia and to further analyze their respective gene expression patterns have resulted in the observation that microglia display a unique microglia transcriptome demonstrating that microglia are distinct from other macrophage populations (Beutner et al., 2013). The establishment of this microglia-specific gene expression profile in mice is restricted to the first postnatal weeks and includes distinct microglia genes such as *Olfml3*, *Tmem119*, *Hexb* and *Fcrls*, which indicate microglia maturation (Bennett et al., 2016). Butovsky et al. (2014) have demonstrated that this maturation is dependent on the presence of Transforming growth factor beta 1 (TGFβ1). Using an elegant transgenic mice approach to overcome the lethal inflammatory phenotype of TGFβ1 mutant mice, this seminal study demonstrated that lack of TGFβ1 in the CNS results in decreased microglia numbers and loss of the microglia-specific gene expression signature. However, the molecular mechanisms of microglia maturation are likely to be more complicated and may not entirely rely on CNS-specific mediators. Recently, the contribution of gut microbiota has been demonstrated to regulate microglia maturation and functions in the CNS (Erny et al., 2015).

Upon establishment of the blood-brain-barrier (BBB), microglia undergo a constant self-renewal in order to maintain their population and it has been shown that no progenitor recruitment from blood circulation is contributing to this maintenance of microglia numbers throughout the lifetime (Ajami et al., 2007). Next to proliferation, microglial apoptosis contributes to maintaining the turnover of microglia in the adult CNS (Askew et al., 2017). A challenging central question is whether microglial self-renewal is the result of asymmetric cell division of microglial precursors in the CNS. Using selective CSF1R inhibitors, Elmore et al. (2014) were able to show that microglia could be effectively depleted (90% depletion) from the CNS. Surprisingly, a complete repopulation was observed and was mediated through a proliferation of nestin-positive precursors, which finally differentiated into microglia (Elmore et al., 2014). These results suggest that the microglia turnover in the adult CNS is mediated from a yet unknown precursor cell population or even from a not yet located stem cell niche. It will be of utmost interest to further characterize the endogenous microglia replenishment in the healthy and diseased CNS.

## Microglia Activation

The paradigm that microglia retain a resting state under non-pathological conditions and only react to endogenous and exogenous inflammatory stimuli has been critically evaluated by Nimmerjahn et al. (2005). Using 2-photon imaging, the authors have shown that microglia are constantly surveying their microenvironment in order to rapidly react and migrate towards impairments such as neuron death, BBB leakage or extracellular ATP accumulation (Nimmerjahn et al., 2005). This observation led to the conclusion that microglia might have important functions under physiological conditions. Recent reports have contributed to broaden our understanding that microglia capacities in the CNS include several essential features such as synaptic pruning (Stevens et al., 2007; Paolicelli et al., 2011; Hoshiko et al., 2012; Schafer et al., 2012), influencing functions of activated and/or over-activated neurons (Panatier and Robitaille, 2012), supporting cortical neuron survival (Ueno et al., 2013), shaping axonal projections (Pont-Lezica et al., 2014; Squarzoni et al., 2014), synapse formation during learning in the adult CNS (Parkhurst et al., 2013) as well as maintenance of synaptic functions in the mature retina (Wang et al., 2016). Although many of the abovementioned functions are mediated during the embryonic and postnatal development of the CNS, microglia support neuronal circuits and, thus, important neurological CNS functions throughout adulthood and aging.

Under pathological conditions microglia rapidly change their morphology and adopt activation states in order to adequately react to the activation-causing stimuli. Microglia proliferation has been reported after traumatic CNS injuries (Streit et al., 1999) and degenerative pathologies including optic nerve lesion (Wohl et al., 2010), AD (Kamphuis et al., 2012), prion disease (Gómez-Nicola et al., 2013), PD (Machado et al., 2016) or ischemia (Li et al., 2013).

Based on the nature of distinct microglia stimuli, pathogen-associated molecular patterns (PAMPs) and danger-associated molecular patterns (DAMPs) have been categorized and are detected by microglial receptors including Toll-like receptors (TLRs), NOD-like receptors (NLRs), RIG-like receptors (RLRs), AIM2-like receptors (ALRs) as well as C-type lectins (Kigerl et al., 2014). In analogy to macrophages, which display remarkable plasticities in response to distinct environmental triggers (Mosser and Edwards, 2008), microglia activation has been subdivided into M1-like and M2-like activation states (Prinz and Priller, 2014). Although M1- and M2-like microglia polarization can be sufficiently induced *in vitro* by using Th1 cytokines such as IFNγ (Zhou et al., 2015) and Th2 cytokines such as IL4 (Zhou et al., 2012), these distinct activation patterns do not seem to be applicable *in vivo* (Ransohoff, 2016). Moreover, macrophage activation patterns classified as M1 and M2 further needs thorough revision due to increasing knowledge about distinct macrophage stimuli (Martinez and Gordon, 2014). With regard to their unique molecular signatures, resident microglia have to be distinguished from infiltrating monocytes/macrophages in terms of gene expression patterns, surface receptors and functions, respectively. Especially under pathological conditions microglia and macrophages display distinct functional properties. Infiltrating monocytes are able to contribute to the resident microglia pool during experimental autoimmune encephalomyelitis (EAE) and have been demonstrated to trigger EAE progression in this context (Ajami et al., 2011). Moreover, T-cell mediated macrophage activation seems to be essential for inflammatory demyelination processes observed during EAE (Yamasaki et al., 2014). Similar observations for monocyte/macrophage contribution to neuroinflammation and subsequent neurodegeneration has been reported for generalized seizures an animal model for epilepsy (Varvel et al., 2016). Of note, inflammatory M1 macrophages entering the aged brain due to BBB impairments have been demonstrated to negatively regulate synaptic functions and LTP formation in the hippocampus (Costello et al., 2016).

Recently identified microglia-specific genes such as *Tmem119* (Bennett et al., 2016) or *Fcrls* (Butovsky et al., 2014) and/or sophisticated microglia isolation protocols (de Haas et al., 2007) can be used in order to identify region-specific microglial differences. Differences in microglial surface receptor expressions (de Haas et al., 2008) and gene expression patterns (Doorn et al., 2015) have been reported and suggest the existence of different functional distinct microglia subpopulations in the CNS. This microglial diversity may be responsible for regionally localized homeostatic functions and might further underlie region-specific sensitivities to microglial dysregulation and involvement in age-related neurodegenerative processes (Grabert et al., 2016).

## Aging Microglia: Priming, Functions and Phenotypes

In healthy rodents, microglia make up 5%–12% of all CNS-specific cells. However, the distribution is diverse and some brain areas display higher densities of microglia (Lawson et al., 1990). Interestingly, the nigrostriatal system including substantia nigra (SN) and the caudate putamen (CPu) show significantly higher microglia densities than adjacent brain regions (Sharaf et al., 2013). Similar observations have been made in humans where microglia represent 0.5%–16.6% of all cells in the brain parenchyma. Moreover, the numbers of microglia in the white matter are higher compared to gray matter (Mittelbronn et al., 2001). Several studies have tried to address the age-related changes in microglia numbers in different species with diverse results. Whereas no obvious changes in Iba1^+^ microglia numbers have been observed in the aged rat hippocampus (VanGuilder et al., 2011), reductions in microglia numbers were detected in the aged nigrostriatal system and cerebral cortex (Sharaf et al., 2013). However, in aged rhesus monkeys (25–35 years) the numbers of microglia increased and the cells displayed heterogeneous intracellular inclusions indicative of increased phagocytosis and reduced capacity to digest engulfed particles (Peters et al., 1991). Dystrophic changes in microglia have been detected in aged individuals and were much more prevalent in older subjects (68-year-old) than in the younger ones (38-year-old; Streit et al., 2004). Interestingly, it has been described that dystrophic or senescent microglia might undergo age-dependent degeneration and are believed to lose their neuroprotective functions, thereby, contributing to the age-dependent onset of sporadic AD (sAD; Streit et al., 2009). Altered microglia morphology and reduced arborization have been reported in the human brain during aging and in AD patients (Davies et al., 2016). It remains unclear whether the observed morphological changes are signs of microglia degeneration and a recent study suggests that the reported microglia dystrophy might reflect age-related cytoskeleton alterations (Tischer et al., 2016). Interestingly, direct Tau uptake by microglia has been reported (Bolós et al., 2016) and is enhanced by anti-tau antibodies in an Fc-receptor-dependent manner (Luo et al., 2015). Moreover, hyperphosphorylated Tau was detectable in aged common marmoset brains and was present in dystrophic microglia suggesting that clearance of pathological protein aggregates might foster microglia dystrophy (Rodriguez-Callejas et al., 2016).

Due to the unique nature of microglia and especially due to their self-renewal capacity, a telomere shortening was hypothesized during aging of microglia. Whereas cells with high proliferative potential, such as stem cells or cancer cells have increased telomerase activity in order to maintain telomere length, somatic cells with limited replication potential possess less telomerase activity (Satyanarayana et al., 2004; Blasco, 2007). Indeed, telomere shortening has been reported in cultured microglia (Flanary and Streit, 2004) and was associated with dementia in human AD brain samples (Flanary et al., 2007). However, it seems that mouse microglia do not develop dystrophy and telomere shortening under normal conditions due to the limited lifespan of mice. Transgenic approaches, such as the Ercc1 mutant mice, a DNA repair-deficient mouse model that displays features of accelerated aging in multiple tissues including the CNS have contributed to the understanding how aging might affect microglia functions. In Ercc1 mutant mice, microglia display hallmark features of priming and increased responses to systemic lipopolysaccharide (LPS) exposure as reflected by cytokine expression and phagocytosis (Raj et al., 2014). Mice lacking the telomerase RNA component (TERC) are characterized by an accelerated aging phenotype associated with enhanced microglia activation in response to LPS and a subsequent immune cell infiltration upon BBB dysregulation (Raj et al., 2015). These results indicate that microglial aging result in functional impairments and increased microglia activation, which is probably involved in the onset and/or progression of neurodegeneration.

Age-dependent microglia activation has been described in aged rodents (Perry et al., 1993), rats (Ogura et al., 1994), humans (Streit and Sparks, 1997) as well as non-human primates (Sheffield and Berman, 1998). Hallmarks of age-dependent microglia activation are increased expression of MHCII (Henry et al., 2009; VanGuilder et al., 2011), CD68 (Wong et al., 2005; Griffin et al., 2006) as well as increased levels of TLRs (Letiembre et al., 2007). Aged microglia have been characterized by the presence of lipofuscin inclusions, reduced processes complexity and increased expression pro-inflammatory (TNFα, IL1β, IL6) and anti-inflammatory (IL10, TGFβ1) cytokines. After LPS challenge aged microglia exhibit increased expression of TNFα, IL1β, IL6 and IL10 (Sierra et al., 2007). Furthermore, increased expression of macrophage inflammatory protein (MIP)1α, MIP1β and RANTES in different brain regions of aged mice have been detected (Felzien et al., 2001). In general, the reactivity of microglia upon stimulation seems to be increased during aging, which is further reflected by enhanced microglia activation in aged mice after injection of activating cytokines IL1β and IL12 to the hippocampus (Lee et al., 2013). This phenomenon has been described as microglia priming and TLR2, TLR3 and TLR4 seem to be essential to prime microglia but not astrocytes for ATP-dependent interleukin-1β release (Facci et al., 2014). Microglia priming induces a highly conserved transcriptional signature with aging- and disease-specific aspects (Holtman et al., 2015), which is dependent on High mobility group box 1 (HMGB1). HMGB1 mediates the neuroinflammatory priming in the aged CNS and inhibition of HMGB1 functions appears to desensitize aged microglia to an immune challenge, thus preventing exaggerated behavioral and neuroinflammatory responses following microglia stimulation (Fonken et al., 2016).

Expression levels of the complement genes, C3 and complement factor B (CFB), both of which being previously associated with age-related macular degeneration (AMD), increased during aging suggesting that senescent retinal microglia may contribute to complement dysregulation during disease pathogenesis and progression (Ma et al., 2013). Although these studies suggest an inflammatory microglia phenotype in aged mice, RNA sequencing revealed that aged microglia display decreased expression of genes associated with endogenous ligand recognition and upregulated genes associated with microbe recognition and host defense. Most interestingly, aged microglia presented increased expression of genes related to neuroprotection and neurorestoration (Hickman et al., 2013).

Among the functional impairments of aged microglia, a reduced capacity to engulf amyloid-β fibrils (Floden and Combs, 2011) and reduced chemotaxis, process motility and migration towards laser-induced injury and extracellular ATP (Damani et al., 2011) have been described. Moreover, cultured microglia from aged mice show a stronger reaction upon ATP-triggered activation, which is characterized by increased nitric oxide (NO) and TNFα release (Lai et al., 2013). Recently, it has been reported that myelin pieces are gradually released from aging myelin sheaths and are subsequently cleared by microglia. This myelin fragmentation increased with age and led to the formation of insoluble, lipofuscin-like lysosomal inclusions in microglia contributing to microglial senescence and immune dysfunctions in aging mice (Safaiyan et al., 2016). Age-dependent microglia dysfunctions might be further enhanced by loss of endogenous regulation of microglia functions and activation states. TGFβ1 has been demonstrated to promote quiescence of microglia *in vitro* (Spittau et al., 2013) and *in vivo* (Butovsky et al., 2014) and is a promising candidate to regulate microglia activation states. Moreover, TGFβ1 inhibits IFNγ-induced microglia activation and degeneration of midbrain dopaminergic (mDA) neurons (Zhou et al., 2015) and induces microglia-mediated engulfment of apoptotic cells via induction of microglial Mfge8 expression (Spittau et al., 2015). Age-dependent impairment of TGFβ1 signaling was lately described to reduce the protective functions of microglia promoting cytotoxic activation and potentiating microglia-mediated neurodegeneration (Tichauer et al., 2014).

Although most of the abovementioned studies have been performed in rodents, recent studies indicate that microglia priming and age-dependent microglia activation is also detectable in the human CNS. *In vivo* imaging using (R)-[(11)C]PK11195 and positron emission tomography revealed that activated microglia appear in several cortical and subcortical areas during healthy aging (Schuitemaker et al., 2012). Further, glial-specific genes shift their regional expression patterns during aging and especially microglia-specific genes globally display increased expression during aging (Soreq et al., 2017). Taken together, priming of microglia and age-related changes in microglia functions and activations are likely to be involved in the development and progression of neurodegenerative diseases such as AD and PD.

## Aging and AD and PD

The most frequent neurodegenerative diseases are AD and PD, both of which sharing a common risk factor namely aging. AD is characterized by progressive cognitive impairments and behavioral disturbances. Neuropathological hallmarks such as neuron death, dystrophic neurites, synapse loss, amyloid plaques and neurofibrillary tangles are accompanied by reactive gliosis (Serrano-Pozo et al., 2011). Basically, AD can be categorized into two distinct subtypes: sAD and familial AD (fAD). Whereas sAD represent a polygenic disorder, fAD is linked to mutations and polymorphisms in genes such as Amyloid precursor protein (APP), Presenilin 1 (PSEN1) or Presenilin 2 (PSEN2) and account for 5%–10% of all AD cases (Bagyinszky et al., 2014). PD is characterized by the progressive loss of mDA neurons in the SN pars compacta and the subsequent reduction in dopamine levels in the basal ganglia, which results in classical movement disturbances such as akinesia, rigidity and tremor. Similar to AD, PD cases can be divided into sporadic PD (sPD) and familial PD (fPD), the latter of which represents approximately 20%–25% of all PD cases. A common hallmark of sPD and fPD is the presence of intracellular inclusions termed Lewy bodies (Goedert, 2001; Jellinger, 2001). α-Synuclein (αSyn) has been identified as a major component of Lewy bodies in sporadic and familial cases and is believed to be the central player in PD etiology (Spillantini et al., 1998). Activated microglia have been described in both pathologies (McGeer et al., 1988) and microglia-mediated neuroinflammatory responses are believed to contribute to disease onset, severity and progression of AD (Heneka et al., 2015) and PD (Block et al., 2007).

In AD mouse models, microglia seem to build up a barrier that prevents neurotoxic effects of proto fibrillar Amyloid-β (Aβ) hotspots around amyloid plaques (Condello et al., 2015). Furthermore, microglia phagocytose Aβ in order to promote clearance of Aβ plaques. Aged microglia display reduced Aβ phagocytosis (Floden and Combs, 2011) and recently it has been demonstrated that Aβ engulfment by aged microglia results in Aβ redistribution rather than in a biophysical degradation of the protein aggregates (Njie et al., 2012). Interestingly, young microglia have been recently demonstrated to restore the amyloid clearance of aged microglia in an elegant *ex vivo* organotypic brain slice co-culturing approach (Daria et al., 2017). In transgenic APP23 mice, exacerbated age-dependent microglia activation and disturbances in microglial cytoskeletal regulations have been described which are high likely to contribute to further neurodegeneration (Janssen et al., 2016). Next to the deteriorated response of aged and primed microglia, complement factor C3 secreted from reactive astrocytes has been reported to interact with the microglial C3a receptor (C3aR) thereby mediating Aβ pathology and neuroinflammation in AD mouse models (Lian et al., 2016). However, lack of C3aR in APP transgenic mice results in decreased, rather than increased, Aβ deposition and C3aR-deficient microglia are more effective in degrading extracellular Aβ (Czirr et al., 2017). Although increased microglia responses as well as impairments of microglia-mediated clearance of Aβ seem to promote the progression of AD, the role of microglia contribution to AD pathology remains unclear. Interestingly, the ablation of microglia in APP transgenic mouse strains crossed with CD11b-HSVTK mice, in which nearly complete depletion of microglia was achieved after ganciclovir application, neither Aβ plaque formation nor amyloid-associated neuron dystrophy was depended on the presence of microglia (Grathwohl et al., 2009). Further studies will be essential in order to broaden our understanding of how microglia contribute to disease onset and progression and a special focus should be given on the role of aged microglia to elucidate the impact of aging on microglial functions in AD.

In PD, marked microglia reactions in the human SN have been observed, which are associated with extraneuronal neuromelanin deposits (Beach et al., 2007). Furthermore, human neuromelanin is able to induce microglia-mediated neuroinflammation and neurodegeneration in rats (Zecca et al., 2008) indicating that the release of neuromelanin from degenerating mDA neurons is a potent trigger for microglia activation. In rodents, toxin-based models, such as 1-methyl-4-phenyl-1,2,3,6-tetrahydropyridine (MPTP) are employed to understand the contribution of microglia to onset and progression of mDA neuron degeneration (Machado et al., 2016). Although the contribution of microglia-mediated neuroinflammation has been clearly demonstrated (Block et al., 2007; Machado et al., 2016), the impact of aging is often ignored in these studies. Interestingly, microglia priming increases the response to the herbicide paraquat and, thus resulting in increased mDA neurodegeneration (Purisai et al., 2007) and aged monkeys display stronger and persistent microglia reactivity after MPTP application indicating microglial involvement in age-dependent degeneration of mDA neurons (Kanaan et al., 2008). It is high likely that age-dependent microglia priming involves epigenetic modifications as reported by Tang et al. (2014). The authors demonstrated that histone H3K27me3 demethylase Jumonji domain containing 3 (Jmjd3) is essential for M2-like microglia activation. Inhibition of Jmjd3 resulted in exacerbation of MPTP-induced mDA degeneration and aged mice displayed reduced Jmjd3 expression and increased H3K27me3 suggesting an age-dependent switch in microglia activation phenotypes (Tang et al., 2014). The effect of aging on the severity of MPTP-induced neurodegeneration is further supported by a study using Senescence-accelerated mouse prone 8 (SAMP8), a mouse line with early onset of senility which presents increased microglia activation and increased neurodegeneration after MPTP intoxication (Liu et al., 2010). Next to toxin-based models for PD, αSyn transgenic mice are often used to understand how αSyn aggregates contribute to neuroinflammation and neurodegeneration in PD. Non-aggregated αSyn is able to trigger TLR-mediated immune responses of microglia, a phenomenon that might contribute to the onset of sporadic and/or familial αSyn-related PD forms (Roodveldt et al., 2013). Notably, telomere shortening has been shown to accelerate αSyn pathology, which is linked to limited microglia function in the brainstem (Scheffold et al., 2016). This observation might be explained by age-dependent microglia deficits. Indeed, isolated microglia from adult mice display phagocytosis impairment of free and exosome-associated αSyn oligomers associated with enhanced TNFα secretion (Bliederhaeuser et al., 2016). Taken together, the contribution of aged microglia to the progressive nature of PD is most likely and the fact that the nigrostriatal system displays a high density of microglia (Sharaf et al., 2013) further supports the hypothesis that microglia are involved in PD pathogenesis. However, the molecular and functional changes of aged microglia are only partially understood and their contribution to neurodegeneration and neuroinflammation in aged individuals need to be further addressed in future studies.

## Conclusion

**Figure 1:**
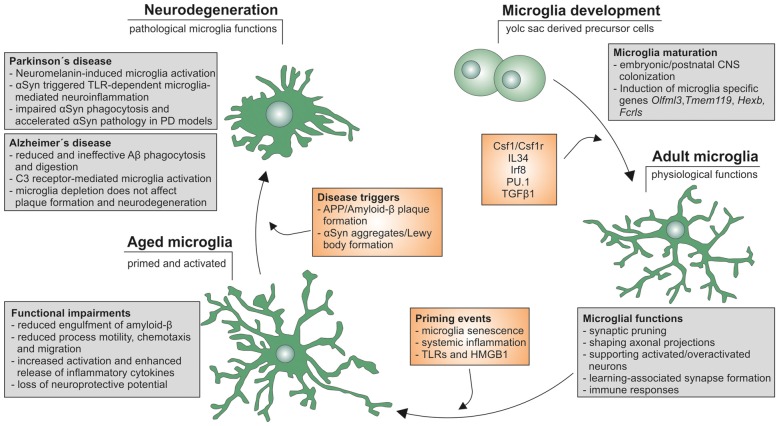
Microglia life cycle. Schematic summary of microglial development, maturation as well as aging and the functional changes influencing onset, severity and progression of neurodegeneration in Alzheimer’s disease (AD) and Parkinson’s disease (PD).

Aging has been clearly demonstrated to affect microglia functions and activation states *in vitro* and *in vivo* and it further appears that the unique nature of microglia contributes to their age-dependent functional impairment. Moreover, the onset, severity and progression of neurodegenerative diseases such as AD and PD are influenced by aging and aging-associated changes in microglia functions (Figure 1). However, it is not clear whether aged microglia are responsible for the exacerbation of neurodegeneration in aged individuals or whether aged neurons itself are more prone to degenerative cues. Furthermore, the contribution of systemic age-dependent changes, such as obesity and metabolic diseases like diabetes, are likely to affect microglia and neuroinflammatory responses. Overall, the effect of aging on microglia needs to be further analyzed in order to better understand the molecular mechanisms underlying age-related changes in microglia phenotypes and functions.

## Author Contributions

BS wrote the manuscript.

## Conflict of Interest Statement

The author declares that the research was conducted in the absence of any commercial or financial relationships that could be construed as a potential conflict of interest.
